# Telemonitoring Parkinson’s disease using machine learning by combining tremor and voice analysis

**DOI:** 10.1186/s40708-020-00113-1

**Published:** 2020-10-22

**Authors:** Md. Sakibur Rahman Sajal, Md. Tanvir Ehsan, Ravi Vaidyanathan, Shouyan Wang, Tipu Aziz, Khondaker Abdullah Al Mamun

**Affiliations:** 1 Department of Computer Science and Engineering, United International University, Dhaka, Bangladesh; 2 Advanced Intelligent Multidisciplinary Systems Lab (AIMS Lab), Institute of Advanced Research, United International University, Dhaka, Bangladesh; 3 Department of Mechanical Engineering, Imperial College London, London, UK; 4 Institute of Science and Technology for Brain-inspired Intelligence (ISTBI), Fudan University, Shanghai, People’s Republic of China; 5 Functional Neurosurgery and Experimental Neurology Group, University of Oxford, Oxford, UK

**Keywords:** Parkinson’s, Tremor, Accelerometer, Machine-learning, Telemonitoring

## Abstract

**Background:**

With the growing number of the aged population, the number of Parkinson’s disease (PD) affected people is also mounting. Unfortunately, due to insufficient resources and awareness in underdeveloped countries, proper and timely PD detection is highly challenged. Besides, all PD patients’ symptoms are neither the same nor they all become pronounced at the same stage of the illness. Therefore, this work aims to combine more than one symptom (rest tremor and voice degradation) by collecting data remotely using smartphones and detect PD with the help of a cloud-based machine learning system for telemonitoring the PD patients in the developing countries.

**Method:**

This proposed system receives rest tremor and vowel phonation data acquired by smartphones with built-in accelerometer and voice recorder sensors. The data are primarily collected from diagnosed PD patients and healthy people for building and optimizing machine learning models that exhibit higher performance. After that, data from newly suspected PD patients are collected, and the trained algorithms are evaluated to detect PD. Based on the majority-vote from those algorithms, PD-detected patients are connected with a nearby neurologist for consultation. Upon receiving patients’ feedback after being diagnosed by the neurologist, the system may update the model by retraining using the latest data. Also, the system requests the detected patients periodically to upload new data to track their disease progress.

**Result:**

The highest accuracy in PD detection using offline data was $98.3\%$ from voice data and $98.5\%$ from tremor data when used separately. In both cases, k-nearest neighbors (kNN) gave the highest accuracy over support vector machine (SVM) and naive Bayes (NB). The application of maximum relevance minimum redundancy (MRMR) feature selection method showed that by selecting different feature sets based on the patient’s gender, we could improve the detection accuracy. This study’s novelty is the application of ensemble averaging on the combined decisions generated from the analysis of voice and tremor data. The average accuracy of PD detection becomes $99.8\%$ when ensemble averaging was performed on majority-vote from kNN, SVM, and NB.

**Conclusion:**

The proposed system can detect PD using a cloud-based system for computation, data preserving, and regular monitoring of voice and tremor samples captured by smartphones. Thus, this system can be a solution for healthcare authorities to ensure the older population’s accessibility to a better medical diagnosis system in the developing countries, especially in the pandemic situation like COVID-19, when in-person monitoring is minimal.

## Introduction

Parkinson’s disease (PD) is the second most common age-related neurodegenerative disorder (after Alzheimer’s), affecting about 7 to 10 million people worldwide. The disease’s prevalence ranges from 41 people per 100,000 of age below 40 to more than 1900 people per 100,000 of age above 80 [1]. The gradual decaying of the neurons that produce a chemical called dopamine causes abnormal brain activities that result in PD symptoms. Therefore, the rate of newly diagnosed cases generally increases with age, whereas only 4% of people with PD are diagnosed before turning to 50. Studies have found that males are 1.5 times more likely to be affected by Parkinson’s than females [2], which calls for the necessity of a gender-based detection system for better screening.

This disease affects patients’ quality of life, makes social interaction more difficult for them, and worsens their financial condition with extravagant medical expenses [3]. PD causes several motor and non-motor symptoms, which gradually become prominent after different disease progression stages. One of the secondary motor symptoms that people with PD may experience is the change in their speech quality or difficulty speaking in worse cases [4]. However, everyone with PD does not experience the same symptoms. Similarly, all the patients do not develop changes in their speech at the same stage of the disease either [5]. For those who are affected, the voice may get softer, breathier, or hoarse over time. The voice tone may become monotone, lacking the usual ups and downs along with some other problems. As a result of this, the patient gradually finds verbal communication very difficult, and also, people who listen to them need to ask them to repeat the sentences quiet often [6]. In medical terms, these problems are known as dysarthria, hypophonia, tachyphemia, etc.

Another significant indicator of this disease is the tremor [4], which is commonly misdiagnosed as the essential tremor (ET). While both ET and PD are neurological disorders, ET is commonly seen in the middle-aged population; of course, the onset could be during the life span. On the other hand, people after 55–60 years of age are in general affected by PD. The distinguishable characteristics between these two are shown in Table 1 [7] based on which the features could be chosen primarily to detect PD from rest tremor analysis. Like voice degradation, this tremor also makes the patient’s life miserable as the limbs’ involuntary movements hinder them from performing the day-to-day activities comfortably [8].

**Table 1:** Distinction between Parkinson’s tremor (PD) and essential tremor (ET)

Factors	PD	ET
Occurrence	Mostly at rest	Mostly during action
Frequency	Low (4–6Hz)	High (and varying)
Amplitude	High and consistent	Low to high varying
Affected-side	Unilateral	Bilateral

Unfortunately, there is no cure for this disease yet but medication to keep the symptoms under control [9]. Besides, early detection of PD is essential as the treatments such as levodopa/carbidopa are more effective if administered in the early stages of the disease [10]. Besides, non-pharmacologic treatments, such as increased exercise, are also easier to perform in the early stages of PD, which may help slow down disease progression as found by studies. However, this early detection is not always feasible, especially for the rural population in underdeveloped countries where trained neurologists are scarce [11]. In this case, a telemonitoring system could address the problem of early detection, patient monitoring, and providing proper recommendations, including setting up appointments with doctors so that people from remote places could quickly get access to the healthcare they need [12, 13]. To achieve that goal, we have previously proposed a cloud-based telemonitoring framework for supporting PD patients in receiving healthcare service in low resource setting [11]. That system achieved $96.6\%$ accuracy by analyzing only the cloud environment’s voice samples for detecting PD.

In this work, we have incorporated the rest tremor analysis in our cloud-based telemonitoring system to increase the detection accuracy since voice degradation is not pronounced in the same way for all PD patients [5]. Besides, we have used a relatively new feature selection method based on information theory to investigate the effect of gender-based different voice feature sets for male and female patients. The next section will review the previous works in this field, followed by the proposed method described at full length. Materials and methods for the study and the result that followed are presented in "Materials and Method" and "Result" sections, respectively. Finally, this paper is concluded with the analysis of performance, direction of improvement, and our future work in the "Discussion" and "Conclusion" sections.

## Review on the related works

The most common method for the diagnosis of PD symptoms is using the Unified PD Rating Scale (UPDRS) [14], and the motor examinations are being done on the subscale of 0 (absence) to 4 (marked) that is described in the self-explanatory Table 2 for both tremor and speech.

**Table 2:** Assigning UPDRS labels based on tremor and speech characteristics

UPDRS	Tremor	Speech
0	Absent	No problem
1	Slight and infrequently present	Loss of modulation yet understandable
2	Mild and persistent	Loss of modulation with unclear words
3	Moderate and present most of the time	Speech is poorly understood
4	Marked and present most of the time	Most speech is unintelligible

In many studies [15, 16] conducted by researchers, acceleration signals for detection of PD has been investigated for its convenience. Later, some classification systems were designed to differentiate among different kinds of tremors such as PD and ET using machine learning (ML) algorithms [17, 18]. Lyons and Pahwa [19] acquired the data by wearable device and designed a classification system which differentiates essential tremor from Parkinson’s tremor.

Although researchers like Palmes et al. [20] investigated the electromyographic (EMG) signals instead of acceleration signals to identify a given tremor, the data acquisition procedure is not a very comfortable one because of the electrode setups. In the study of Kotsavasiloglou et al. [21] a machine learning model was developed to differentiate healthy subjects and PD patients using the trajectory of horizontal lines drawn by them on a tablet using an electronic pen which is, however, not suitable for collecting data from rural areas in developing countries due to the lack in technological advancement.

Like tremors, numerous studies were conducted on speech analysis of PD patients with a view to the detection of PD and its severity. Work of M. A. Little [22] is one of the most reviewed works which created the database with 22 voice features, which can be found in [23]. In this work, we have used the same data set to compare the performance with similar works found in [24] and [25].

Later, many researchers as like in [26–28] worked on different voice data sets to improve the detection accuracy by feature engineering or applying different classifiers for developing telemonitoring systems. They also investigated different feature selection techniques to reduce dimensionality. However, none of them are known to investigate gender-based models. Since males are more likely to be affected by PD, and the voice features are quite different for males and females, it leaves the questions whether a gender-based detection process would increase the accuracy or not.

## Proposed method

The proposed method jointly uses the *rest* tremor and the *vowel* phonation of the previously *diagnosed* patients with assigned UPDRS labels and other healthy people to train and optimize ML models. The best performing models are then applied to predict and inform the suspected PD patients’ disease status. If PD is detected, the system then assigns a predicted UPDRS level to those patients and recommends them to consult with a trained neurologist. Upon receiving feedback from the patients after a neurologist re-evaluates their condition, the system updates the model by retraining if necessary.

**Fig. 1:**
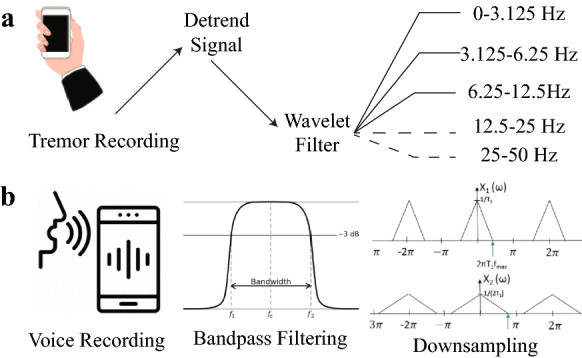
Tremor and voice data acquisition. **a** Raw rest tremor data acquired by the accelerometer is first passed through a detrend filter which is then segmented in different frequency bands using wavelet filter-bank. **b** The vowel phonations captured by the recorder is passed through a band-pass filter which is then downsampled before feature extraction

The system also keeps records of the patient’s status for future reference and requests the patients periodically for uploading new samples for continuous monitoring of disease progression. Simultaneously, the system keeps records of its performance in predicting PD and assigning UPDRS labels accurately. Figure 1 shows the system’s first phase; the data acquisition and pre-processing for telemonitoring PD patients.

### Phase 1: acquisition and pre-processing

#### Tremor data

Rest tremor data of diagnosed PD patients (with UPDRS level determined by trained neurologists), and healthy persons are captured using a smartphone with a built-in 3-axis accelerometer sensor. The data are recorded in an absolute unit ($m/s^2$) and saved in a .txt file, which is then transferred to a laptop for signal processing. Data should be collected from both hands following the directions mentioned in the mobile application (see [29] for details of the data acquisition process) and "Material" section of this paper. However, data from the hand that shows prominent tremors has to be used for classification and detection purposes.

**Fig. 2:**
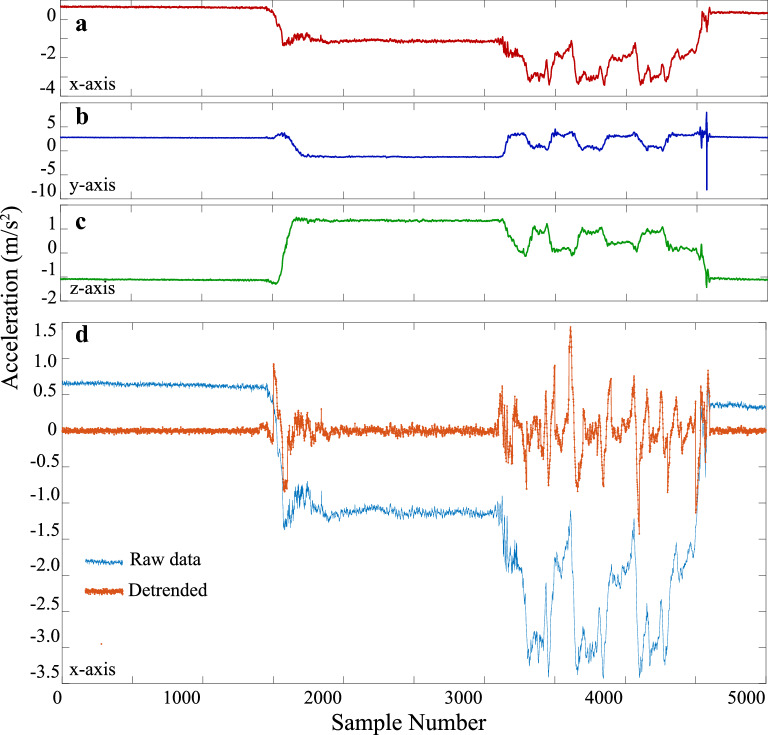
Raw data and detrended data: **a**–**c** x,y and z axis acceleration data with smartphones orientation change during experiment. **d** Detrended x-axis data to remove the effect of DC-offset

The tremor data first goes through a ’detrend’ filter since accelerometer data might have an offset due to the phone’s orientation, i.e., acceleration due to gravity in z-axis data or changing the hand’s position during the experiment. This filtering is performed by windowing all the channels’ data without overlaps and removing the mean acceleration value from the respective windows. This DC offset could also be removed during recording by using alternate coupling (AC) mode. Figure 2 displays the raw 3-axis signal and the effect of detrending. Figure 2a–c shows the effect of changing the orientation of the phone during data recording (see the change in the base level in all the channels). However, detrending pulls back the acceleration values to zero-base value (see Fig. 2d for the detrended data generated from the x-axis) from which we will extract features for classification.

Since the rest tremor has intermittent nature, we used wavelet filtering to have the time-localized resolving capabilities. Furthermore, the frequency ranges of interest (0–3 Hz for healthy people, 3–6 Hz for rest tremor of PD patient, and 6–12 Hz for postural tremor if any [29]) can be easily separated using the wavelet 3-level filter banks (see Fig. 1) when the sampling frequency is 100 Hz (which is most common for the available smartphones). Now the signal at each band is analyzed for target feature extraction.

#### Voice data

Vowel phonations were recorded using a smartphone, keeping it about 8cm away from the patients’ mouth. They were instructed to pronounce the vowel $/{{{\varvec{\alpha }}}}/$ using a single breath for a maximum of 10 s duration while keeping the intensity as stable as possible (see [22] for details of data collection).

The voice samples were first truncated in the time domain as the vowel phonation sound gradually decreases due to the loss of lung pressure at the end. In this way, we maintained the reliability of the signals. Also, the sudden utterance seems to have a high-energy input at the beginning of each recording. Therefore, we discarded the first second and last three seconds to keep the reliable portion of the signal likewise in [22].

Similarly, to remove the noise outside the significant frequency range of human speech (which is 50–8 kHz), we used a band-pass filter with $-\,3dB$ cut-off points at 50 Hz and 8 kHz (f1 and f2 in Fig. 1b) to remove the low- and high-frequency components. The central frequency, fc, is the average of f1 and f2.

Finally, the filtered voice signal was passed through a down-sampler to reduce the sampling frequency to 22.05 kHz $(X_2(\omega ))$ to reduce sample size since the nominal audio recorder sampling frequency, that is 44.1 kHz $(X_1(\omega ))$, is much higher than the Nyquist requirement (16 kHz) to capture voice signal. As voice signal does not have considerable energies in the frequency over 8 KHz, we under-sampled the data to reduce computational power while carefully avoiding aliasing by filtering it beforehand (see the effect of downsampling in the frequency domain from Fig. 1). Also, the signal was normalized since the maximum amplitude does not carry reliable information because the voice intensity could vary from person to person [22], which is not related to PD.

### Phase 2: feature extraction and selection

Based on the qualitative and quantitative characteristics of the rest tremor at different UPDRS levels, as shown in Table 3, we propose some *intuitive* features in addition to those proposed by others in the similar works [29, 30]. The features are listed in Table 4 with the objectives in mind behind choosing them.

**Table 3:** UPDRS label based on the rest tremor characteristics (qualitative and quantitative)

UPDRS	Qualitative		Quantitative	
Labels	Amplitude	Consistency	Max. amplitude	Occurrence in recording
0	Low	Absent	No tremor	No tremor
1	Medium	Intermittent	< 1 cm	≤ 25%
2	Med. or high	Intermittent	≥ 1 cm but < 3 cm	> 25% but ≤ 50%
3	Med. or high	Continuous	≥ 3 cm but < 10 cm	> 50% but ≤ 75%
4	Very high	Continuous	≥ 10 cm	> 75%

**Table 4:** List of features, their focus and ranks in analyzing rest tremor for PD detection

Serial	Feature name	Focusing on	Mean	Variance
1	Average amplitude	Tremor intensity	10	3
2	Peak variation	Intermittent nature	13	11
3	Consecutive peak change	Intermittent nature	14	9
4	Tremor occurrence percentage	Prevalence	7	8
5	Peak location variation	Consistency	5	6
6	Zero crossing rate	Consistency	2	15
7	Maximum power	Energy content	4	1
8	Frequency at max. power	Energy content	12	16

These features are chosen to identify the nature of the amplitude of the tremor and the occurrence pattern. With a window length of 20 samples with a $25\%$ overlap, the features were extracted from all the acceleration axes. The mean and variance are taken to convert it into the feature vectors. For the voice data, however, we used the UCI data repository where all the 22 features were extracted, as shown in Table 5 (see [22] for details of the features). Although we have developed a fully functional system that can extract these features from any vowel phonations, we used this well-known data set for comparing the performance of the proposed system with previously published works.

For feature selection, we used *maximum relevance minimum redundancy* (MRMR) [31] algorithm, which is a feature selection method based on information theory. This algorithm ranks the features based on their mutual information and correlation. The ranks of the tremor and voice features are presented in Tables 4 and 5, respectively.

**Table 5:** Names and description of 22 voice features with their ranks calculated from both (B), only male (M) and only female (F) data

Serial	Feature name and description	Rank (B)	Rank (M)	Rank (F)
1	MDVP: Fo(Hz); *Avg. Fundamental Freq.*	3	2	5
2	MDVP: Fhi(Hz); *Max. Fundamental Freq.*	9	13	2
3	MDVP: Flo(Hz); *Min. Fundamental Freq.*	8	5	21
4	MDVP: Jitter [32]; $(\%)$	17	18	3
5	MDVP: Jitter(abs) [32];	19	22	10
6	MDVP: RAP [32];	22	21	16
7	MDVP: PPQ [32];	13	9	12
8	Jitter: DDP [32];	21	17	18
9	MDVP: Shimmer [32];	11	19	19
10	MDVP: Shimmer(db) [32];	15	10	20
11	Shimmer: APQ3 [32];	20	16	13
12	Shimmer: APQ5 [32];	14	1	15
13	MDVP: APQ [32]	7	14	11
14	Shimmer: DDA [32];	5	20	17
15	NHR; *noise-to-harmonics ratio* [32]	16	12	14
16	HNR; *harmonics-to-noise ratio* [32]	18	8	22
17	RPDE; *recurrence period density entropy* [33]	12	11	6
18	D2; *correlation dimension* [34]	2	3	7
19	DFA; *detrended fluctuation analysis* [33]	1	7	9
20	Spread1; *a non-linear measure* [22]	4	4	1
21	Spread2; *a non-linear measure* [22]	6	6	8
22	PPE; *pitch period entropy* [22]	10	15	4

### Phase 3: model training and optimization

After feature selection, we explored different classifiers and optimized them for maximum accuracy. From the studies of others, it is found that k-nearest neighbors (kNN) and support vector machine (SVM) usually provide the best result in these datasets [24]. Naive Bayes is found to perform well when the number of features is reduced [11]. Therefore, we primarily investigated these three classifiers using the MATLAB ML toolbox and extracted the best online evaluation model after optimization.

The classification was performed with all the features from voice and tremor in the beginning separately. Then we reduced the number of features based on their ranks generated by the MRMR feature selection method to see the effect in the accuracy obtained from the mentioned three ML algorithms.

Finally, we created a test dataset by combining the voice and tremor features to see if accuracy improves from combined decision generated from different modalities. To create each synthetic patient, we concatenated multiple instances of voice and tremor samples of PD positive patients and assigned different name-tags for them. The same was done to create synthetic healthy patients’ dataset. Then this test dataset was evaluated by the optimized models. In this process, we used the majority-vote for PD detection. If at least two of the three models detected PD from a separate analysis of a synthetic patient’s voice and tremor data, the patient is finally reported as PD positive. In case the majority-vote contradicted, for example, PD detected from voice but not from tremor, we fed different instances from the same patient until a unanimous decision is reached from two modalities. A patient is declared as non-PD only when both the voice and tremor analysis returned negative.

## Materials and methods

### Data acquisition protocol

During recording the rest tremor, the patient should sit quietly in a chair, keeping the hands placed on the arms of the chair (not in the lap) with the palm facing down. The feet must be comfortably supported on the floor. The smartphone is to be attached to the hand’s back for recording data for 10 s with no other directives. Rest tremors should be assessed separately for both hands, and only the maximum amplitude that is seen at any time during the recording period has to be taken as the final *amplitude* rating. For tremor *consistency* recording, the collection of the data could start from the beginning of the patient examination (during interviews and questionnaire filling), so that data of several minutes are at hand besides the purposefully recorded 10 s tremor data for assessing maximum amplitude. In that case, the body part can be kept variously at rest, keeping the smartphone attached to the hand, and the hand is placed comfortably on support like the thighs or arm of the chair (but not both hands in finger locked position).

The smartphone has to be stationed at a distance of about 8 cm from the mouth of the patient to collect the vowel phonation. The patient has to sit relaxed and take a deep breath before uttering the vowel sound. The patient must be instructed to keep the intensity as consistent as possible during the data recording of 10 s. Patients can attempt multiple recordings, but try not to have a break in an utterance in the middle of it as data from the beginning and end will be truncated to analyze the middle portion of every recording.

### Data source

Tremor data were collected at Hazrat Rasoul Akram Hospital of Tehran, under the supervision of expert neurologist [35]. Fifty-two patients volunteered to provide data for the study. We used this data set to evaluate our system (its feature generation, selection, and detection accuracy) for comparative result analysis.

On the other hand, the extracted voice features were collected from [23] created with the consent of the patients involved in the study. We used this data set for evaluating the performance of the proposed system with the previously published works. The details of the data collection procedure and the description of the features can found in [22].

We used the signal processing and machine learning toolbox of MATLAB2019b in a laptop computer to perform signal processing, analysis, and classification on the offline data sets mentioned in this work. Meanwhile, a mobile application is developed to acquire tremor and vowel phonation data from people at remote places that can send those data to the server where a cloud-based ML system is created for real-time data analysis.

## Result

### Results on voice data

**Fig. 3:**
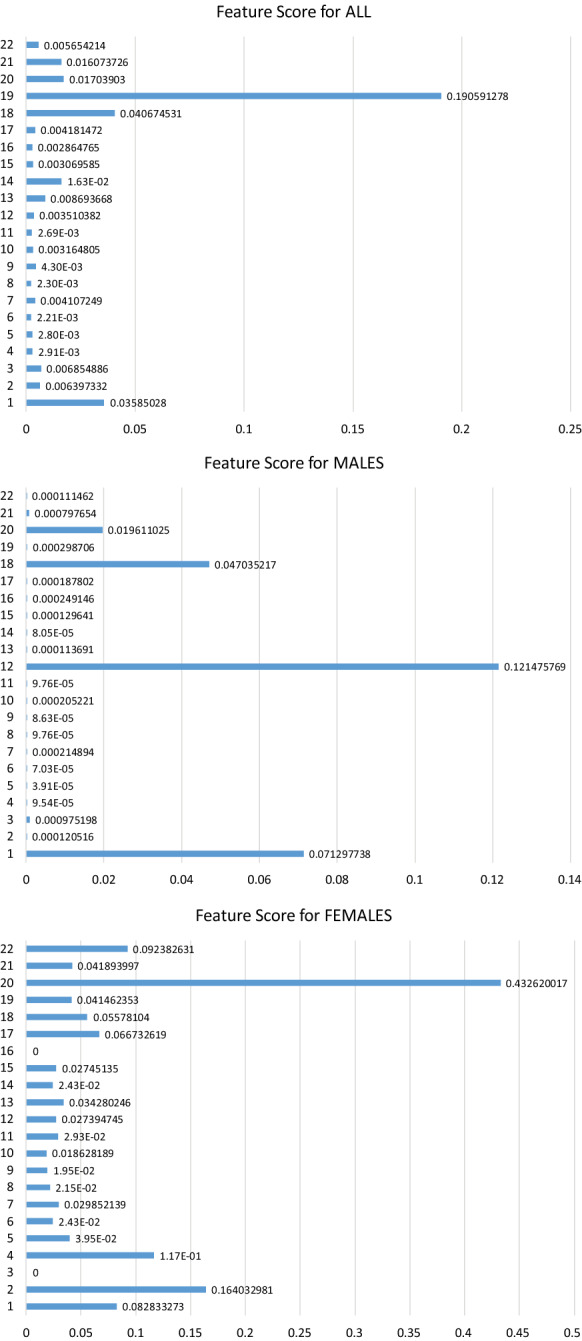
Relative weights of all 22 features in detection of PD for both and only male and female cases

The MRMR feature selection method’s application resulted in ranking the features differently for only males, females, and all together. Table 5 shows the names of 22 voice features, and their ranks for both (B), only males (M), and only females (F) training data sets. However, for visual aid, Fig. 3 displays the relative weights of each of the features in different data sets. Using all the features, top 10 and top 5 ranked features separately with different classifiers (kNN, SVM, and naive Bayes) at tenfold cross-validation, the accuracy, sensitivity, and specificity are presented in Table 6.

As we have found that for the male data set, the highest accuracy using all the features was found in kNN having k = 57, Distance metric: ’Correlation’ and distance weight: ’squa

**Table 6:** Accuracy, specificity and sensitivity from kNN, SVM and naive Bayes using voice features

ML	Feat.	Male			Female			Both		
Algo.	No.	Accu.	Spec.	Sens.	Accu.	Spec.	Sens.	Accu.	Spec.	Sens.
kNN	22	98.3	89.0	100	95.9	93.0	98.0	93.7	93.7	94.6
	10	93.0	83.0	96.0	94.6	97.6	99.6	92.1	87.5	93.6
	5	93.9	89.0	96.0	94.6	93.0	95.0	93.1	86.4	94.6
SVM	22	92.3	88.0	96.4	95.9	93.3	97.7	89.9	85.0	93.0
	10	93.9	88.0	97.0	94.6	93.3	95.5	90.5	89.5	93.6
	5	84.3	82.0	90.0	94.6	92.0	96.0	90.5	89.0	94.0
Naive Bayes	22	87.2	84.0	92.0	77.0	73.3	77.3	74.6	72.5	75.0
	10	90.4	87.0	94.0	86.5	83.3	90.0	80.1	77.0	87.0
	5	94.8	88.0	96.5	87.8	85.0	92.0	81.0	80.5	89.5

red inverse’ during optimization. SVM used a quadratic kernel, and naive Bayes used a Gaussian kernel for maximum accuracy at 22 features. However, as we have reduced to the top 10 features for classification, the accuracy for kNN decreased while SVM and naive Bayes increased (see Table 6). With more dimensionality reduction, naive Bayes reached its peak while others dropped to the least. In this male data set, the highest specificity and sensitivity were $89\%$ and $100\%$, respectively, for kNN.

For female voice samples, accuracy with all 22 features decreased in kNN and naive Bayes, but increased in SVM compared to male voice samples, as shown in Table 6. Parameters for kNN were found as k = 3, ’correlation distance metric with equal weight. SVM and naive Bayes used ’quadratic’ kernel and ’Epanechikov’ kernel, respectively. Similarly, like before, reducing the number of features improved the accuracy for naive Bayes; however, not up to the mark. On the other hand, accuracy for kNN and SVM decreased only slightly, unlike the male data set. This time the highest specificity and sensitivity were $97\%$ and $99\%$, respectively, for kNN with the top 10 features.

Finally, training on both male and female data together resulted in lower accuracy in all the classifiers than independent learning. kNN was optimized with distance metric ’Minkowski’ with ’inverse’ distance weight and k = 2. SVM used ’cubic’ kernel, and naive Bayes used box kernel to reach the result shown in Table 6 using all the features. Reducing the features did not change the accuracy for kNN and SVM that much; however, the accuracy for naive Bayes improved as like before. The highest specificity and sensitivity were $93.7\%$ and $94.6\%$, respectively, in kNN.

### Results on tremor data

From the analysis of tremor data, we got a maximum of $98.5\%$ accuracy in determining PD vs. non-PD (2-level classification) using kNN with the top 8 features (see Table 7 for the rank of the features). It should be noted that each feature category includes three feature values generated from each of the axes. Therefore, the total number of features is $16\times 3$ or 48 if all the extracted features are considered, which is not suitable for practical application. Therefore, the top 8 ranked feature categories (that gives 24 feature values when all three channels are combined) identified by the MRMR feature selection process were used in the final result generation. For SVM and naive Bayes, the accuracy was $96.8\%$ and $91.6\%$, respectively, as shown in Table 7. The highest specificity and sensitivity were $100\%$ and $94.0\%$, respectively, for kNN.

**Table 7:** Classification accuracy, specificity and sensitivity for the rest tremor analysis

Used	2-level classification			5-level classification		
Classifier	Accuracy	Specificity	Sensitivity	Accuracy	Specificity	Sensitivity
kNN	98.5	100	94.0	90.5	96.0	87.5
SVM	96.8	98.0	92.0	87.0	91.0	86.5
Naive Bayes	91.6	95.5	89.5	77.0	72.0	81.5

However, when we went for 5-level classifications (UPDRS labels 0 to 4), the accuracy was found to be $90\%$ using kNN while others exhibit below $90\%$ (see Table 7 for details). The highest specificity and sensitivity were found to be $96.0\%$ and $87.5\%$, respectively, for kNN this time.

### Results on combined data

Creating a combined data set by associating the tremor and voice data of the PD patients and the healthy persons, we found that the combined accuracy of detection (PD and Non-PD) becomes $99.8\%$, which is obtained by applying the ensemble averaging on the majority-vote for decision-making regarding patient status.

For this investigation, the tremors analysis’ selected features were concatenated with the voice features, and patient profiles were created with multiple samples/instances for each patient. This group was assessed by the optimized algorithms using voice and tremor samples separately. A patient was reported to have PD only when 2 out of 3 algorithms returned PD positive results from both modalities. In case both of the modalities did not agree for a particular PD patient, the system assessed other samples corresponding to that patient until a decisive result is obtained from the majority vote. Each time the patient was given a score out of three based on the number of models which returned PD positive result. A patient is finally reported as PD positive if the average score from the tests on all the instances exceeded the threshold value of $60\%$ (since 2/3 would have been the threshold for majority-vote in this case, we chose this value as the common threshold whether contraction occurred or not). Otherwise, the person was reported as PD negative.

## Discussion

As a pilot project, the offline datasets’ results support the proposed system’s effectiveness in a pandemic situation like COVID-19 that we are currently facing. The remote monitoring of PD patients can be performed by analyzing their vowel phonations and tremor data collected by smartphones. We will soon begin the clinical trial to assess the system in a real-life situation.

From what we have found by analyzing the voice data is very much interesting since the accuracy varied from male to female because of choosing different feature sets based on gender influence. Some of the features introduced by Max A. Little [22] seemed to have higher ranks than the conventional ones, as we found in the case of standard data (both male and female). Tsanas [25] later investigated the same with the help of correlation coefficients for identifying more informative features. However, some conventional features even performed better in the gender-based analysis (see Fig. 3 for relative weight after feature ranking) as some intrinsic properties of the voice vary differently from male to female PD. The result obtained in [24] is almost the same as what we found in this work while using both male and female data set without distinction, which supports the assertion of accuracy improvement by using separate feature sets for male and female data sets.

Moreover, for the female data set, the reduction of features did not affect the accuracy much compared to the male data set. On the other hand, the male data set showed the highest accuracy using conventional features. Therefore, the scope of more than one classifier is evident in the detection system if the patients are willing to share the gender information. The study of different age groups might come with similar insight, which will be included in our future work. In that case, multiple models will be trained, and the better-performing ones will be used for testing patients of different age groups if necessary.

As for the accuracy obtained using the tremor data in this study, 2-level classification results are very much appreciable from the practical point of view. However, the accuracy degrades for 5-level classification since the difference between UPDRS levels 1 and 2 and UPDRS levels 3 and 4 is very subtle (see Table 3 for clarity). Although it was shown in [35] that the result could be improved by taking smaller feature sets and using the naive Bayes algorithm, this holds for small data sets only with fewer outliers. As our system is designed to receive data recorded by the patients’ family members, mostly from remote locations, we focused on a relatively more robust system. Therefore, kNN and SVM, which perform better in large data sets, were our primary interest. However in this study, UPDRS levels were not determined from the combined data set as the patients were different, and they were not assessed with compatible labeling schemes. For the system developed in this work, we have adopted the labeling scheme of Movement Disorder Society (MDS-UPDRS) for voice disorder and rest tremors (details can be found in [36]). When the system is evaluated in a clinical trial, the neurologists will be following this labeling protocol.

It is well-known that SVM takes care of the outliers better than kNN. Outliers could be a challenging issue when this system is implemented practically for two reasons. Firstly, untrained professionals could collect the data to send to the cloud system while a trained neurologist might assess the PD status. Secondly, the UPDRS level assignment criteria may vary for adopting different guidelines. On the other hand, kNN outperforms SVM when the data set is much larger than the number of features, which is also true for the proposed system as the number of old populations and the PD patients are overgrowing in developing countries. With the continually growing training data, the system will need to update the models periodically to improve its performance. For these reasons, the majority-vote process was utilized before reporting the patient’s status in the proposed system.

The inaccuracy in the detection of PD might have some possible reasons that include the variability in the severity of PD across patients, the sensor’s intrinsic uncertainty, and the fact that some patients may consciously or subconsciously suppress their tremors during the experiment. In previous studies, [37] and [38], PD tremor is considered and reported as a single frequency signal, and the fundamental frequency ranges between 4 to 12 Hz. However, the results in [30] showed that PD tremor consists of several harmonics. Therefore the classification accuracy might be improved by incorporating the third stage detailed signal into consideration.

## Conclusion

This paper presents a study on the prospect of combining the tremor and voice data analysis for detecting PD in the remote/underdeveloped areas where trained neurologists are not readily available. Our focus was on utilizing the availability of the latest smartphones with built-in accelerometer and voice recorder sensors from which the collected data can be easily transferred to the cloud-based signal processing and machine learning units. We used the wavelet filter banks to preserve the tremor signals’ time-localized transient nature and extracted useful features for distinguishing different UPDRS levels. On the other hand, we used an information theory-based feature selection method to narrow down the conventional voice features from which easily implementable models are trained. Besides, we have shown the advantage of selecting gender-based different sets of features from voice data to improve detection accuracy.

As for future work, we will incorporate some image processing-based symptoms analysis. For example, palm opening–closing and finger tapping can be analyzed from the video data captured by the same smartphone used for tremor and voice data collection. Also, the accelerometer sensor can be utilized for collecting the gait information during walking of the patient, and gait analysis may offer more reliability in PD detection.

Nevertheless, the result obtained from combining the tremor and voice data in this work implies that this system can identify PD and its stage more reliably than a single-modality detection system. Besides, it can provide recommendations to the patients while updating the patient files with the latest disease progression stage. This collected database can be used upon their consent for population studies on the incidence of Parkinson’s, which are essential to scientists’ understanding of its history, progression, and risk factors. In this way, this system can help healthcare experts design strategies to meet patients’ needs, especially for rural areas where access to a neurologist is minimal.

## Data Availability

The datasets supporting the conclusions of this article are available in the UCI data repository (https://archive.ics.uci.edu/ml/datasets/parkinsonshttps://archive.ics.uci.edu/ml/datasets/parkinsons) and with author of [29].
